# Multidecadal increase in plastic particles in coastal ocean sediments

**DOI:** 10.1126/sciadv.aax0587

**Published:** 2019-09-04

**Authors:** Jennifer A. Brandon, William Jones, Mark D. Ohman

**Affiliations:** Scripps Institution of Oceanography, University of California, San Diego, San Diego, USA.

## Abstract

We analyzed coastal sediments of the Santa Barbara Basin, California, for historical changes in microplastic deposition using a box core that spanned 1834–2009. The sediment was visually sorted for plastic, and a subset was confirmed as plastic polymers via FTIR (Fourier transform infrared) spectroscopy. After correcting for contamination introduced during sample processing, we found an exponential increase in plastic deposition from 1945 to 2009 with a doubling time of 15 years. This increase correlated closely with worldwide plastic production and southern California coastal population increases over the same period. Increased plastic loading in sediments has unknown consequences for deposit-feeding benthic organisms. This increase in plastic deposition in the post–World War II years can be used as a geological proxy for the Great Acceleration of the Anthropocene in the sedimentary record.

## Teaser

Microplastics in ocean sediments increased exponentially from 1945 to 2009, serving as a geological proxy for the Anthropocene.

## INTRODUCTION

An estimated 4.8 million to 12.7 million metric tons of plastic waste enters the ocean every year (*1*). Larger populations produce more waste, and the world population is predicted to increase disproportionately in coastal regions (*2*). As coastal populations grow and synthetic clothing and plastic production increases, effluent-derived fibers are becoming a larger concern in nearshore areas (*2*). Previous studies focusing on buoyant plastics collected at the sea surface have shown a one– to two–order of magnitude increase in abundance of surface microplastic debris in the northeast Pacific from 1972–1987 to 1999–2010 (*3*, *4*), but no significant increase in sea surface plastics in subtropical latitudes of the northeast Atlantic from 1986 to 2008 (*5*). There is a clear need for assessments of longer-term rates of accumulation in coastal ecosystems apart from surface waters. Here, we analyze microplastic particle deposition in coastal ocean sediments off southern California and demonstrate a pronounced and unabated increase in plastic deposition following World War II.

## RESULTS

### The sediment core

**Fig. 1:**
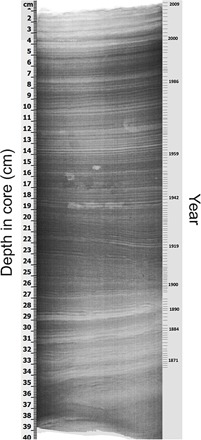
X-radiograph of sediment box core. Chronology was assigned by enumerating individual varve couplets. A bacterial mat 1 to 2 cm thick at the top of the core indicates that surficial sediments were intact.

A box core that sampled Santa Barbara Basin sediments (fig. S1) showed a well-defined annual varved structure (Fig. 1), making it possible to establish a clear chronological sequence. Santa Barbara Basin was chosen because of its unique sediment structure. The high surface productivity in the Santa Barbara Channel, coupled with the restricted water movement due to the Santa Barbara Coastline in the north, the Channel Islands in the south, and the high eastern (230 m) and western (475 m) sill depths, creates an area of anaerobic bottom water, which minimizes bioturbation and allows the preservation of millimeter-scale seasonal laminae couplets, each couplet representing a year (Fig. 1) (*6*). Complete methods are found at the end of this paper.

### Plastics in the sediment core

**Fig. 2:**
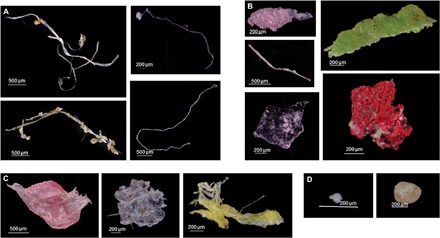
Plastic particles from box core. Examples of (**A**) fibers, (**B**) fragments, (**C**) film, and (**D**) spherical particles.

Plastics were present and visually identified in every 0.5-cm transverse layer of the core (averaging 2.2 years per layer), including in the layers before 1945, before plastic polymers were produced in high quantities or widely used (*1*, *7*, *8*). The year 1945 was also the end of World War II, leading to many societal shifts in production and industry, and is the year denoting the beginning of the Great Acceleration of the Anthropocene (*9*, *10*). Plastic particles were categorized as fibers, fragments, film pieces, and quasi-spherical particles (Fig. 2). Physical characteristics of every particle were recorded, including length, width, color, particle type, and amount of biofouling (fig. S2). The majority of plastics found in the core were fibers, which formed 77% of the particles (fig. S3). The contamination samples, from 1836 to 1945, were more dominated by fibers, at 89.1% of total particles (fig. S3). In post-1945 layers, 67.5% of the particles were fibers (fig. S3). Although previous literature has reported mostly bright-colored fibers and potentially overlooked many neutral-colored fibers (*2*), here the most common fiber color found was white (64.5%). The next most common particle category was fragments, at 14% of the overall particles, although there were many more in the post-1945 samples than in the pre-1945 samples (20.8% versus 5.8%, respectively) (fig. S3). A total of 9.7% of the post-1945 samples were film, compared to 4.9% of the pre-1945 samples. Almost no spherical plastic particles were found in the core (fig. S3).

### Identification of plastics via FTIR spectroscopy

**Fig. 3:**
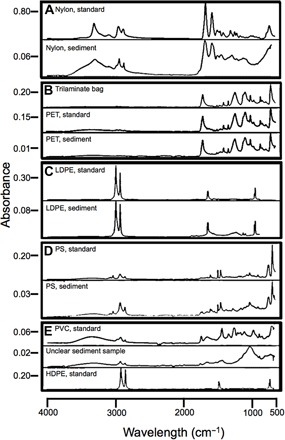
FTIR spectra of plastic standards and sediment samples. PET, polyethylene terephthalate; LDPE, low-density PE; PS, polystyrene; PVC, polyvinyl chloride; HDPE, high-density PE; Unclear sediment sample, unidentified.

Fourier transform infrared (FTIR) identifications of plastic particles (Fig. 3) were sometimes difficult due to small particle size, particularly the small width of fibers, but 87.5% of visually identified plastic particles were definitively or likely plastic polymers, based on matching with standard plastic reference spectra (table S1). The plastic polymers that were identified in the core included polystyrene (PS), polyethylene (PE) including low-density polyethylene (LDPE), polyvinyl chloride (PVC), nylon (polyamide), polyester, PE terephthalate (PET), polypropylene (PP), and the box core liner (which had a distinctive FTIR signature).

### Plastic deposition rates

**Fig. 4:**
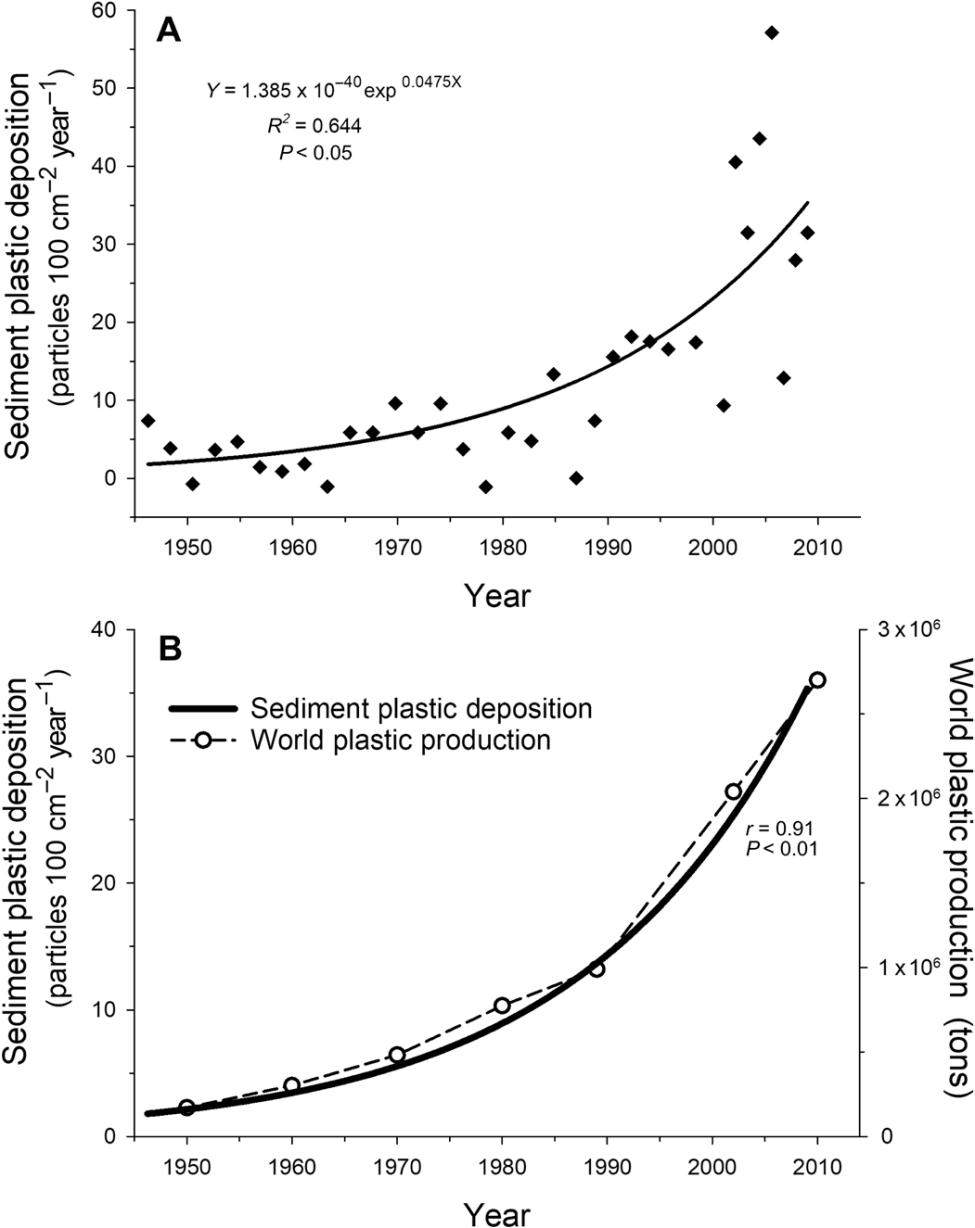
The exponential increase in microplastic deposition in sediment is significantly correlated with the exponential increase in worldwide plastic production over the same time period (1945–2010). (**A**) Total plastic deposition rate over time, corrected for contamination. All four plastic types combined, 1945–2009, with average value from 1836–1945 subtracted. (**B**) Plastic deposition rate in sediment compared to worldwide plastic production, 1950–2010. Worldwide plastic production numbers from PlasticsEurope (*8*).

The plastic deposition rate (Particles*100 cm^−2^ year^−1^) was calculated for the four individual particle types, from 1836 to 2009 (fig. S4). Pieces of core liner, identified via FTIR, were treated separately from other fragments. Fibers dominated the fluxes (fig. S4B). The majority of pre-1945 plastic pieces were fibers of 500 to 1000 μm in length (fig. S2). The average contamination value of 7.8 particles 100 cm^−2^ year^−1^ for all pre-1945 samples was subtracted from all post-1945 samples, yielding the net change in plastic deposition rates over time since 1945 (Fig. 4A). Accordingly, plastic deposition rates in the Santa Barbara Basin from 1945 to 2009 increased exponentially, with an average doubling time of 15 years (Fig. 4A).

### Plastic deposition rates and environmental factors

Residuals of corrected plastic deposition rate of total plastics from the fitted exponential of Fig. 4A show that 1984, 1994, and 2002–2005 had anomalously high plastic deposition rates, and 1978, 1985, 1998, and 2007 were years of anomalously low deposition (fig. S5A). Attempts to relate these anomalies to years of anomalous rainfall, and thus coastal effluent, showed that plastic deposition residuals were not related to residuals of rainfall in either Santa Barbara or Los Angeles County (*P* > 0.05, fig. S5C) (*11*, *12*) or to the Oceanic Niño index (ONI), a measure of El Niño (*P* > 0.05, fig. S5B) (*13*). Much of Santa Barbara Basin sediment is supplied by riverine flow, which is most likely a source of land-based microplastics into the basin. Food web transformations in the highly productive surface waters overlying the Santa Barbara Basin also likely facilitate transport of plastics to the sediment, via ingestion, fecal pellet formation, biofouling, and sinking.

### Plastic fibers and contamination

Because fibers dominate numerically, the particle deposition rate of only fibers, as contrasted with fragments, film, plus spherical particles, is treated separately in fig. S6 (A and B). The deposition rate of both categories of particles treated separately also increased exponentially. The post-1945 rate of increase of combined fragments, films, and spherical particles was not significantly different from the rate of accumulation of fibers alone [analysis of covariance (ANCOVA), *P* > 0.05]. Because these rates do not differ, and the entire core was taken and processed at one time, we have no reason to expect that the processing contamination of plastic fibers in more recent decades of the core would be different than in the pre-1945 time period of the core.

### Plastic deposition rates and societal trends

Multidecadal changes in plastic deposition rates in the Santa Barbara Basin are directly correlated with increases in world plastic production (Pearson’s correlation, *r* = 0.91, *P* < 0.01, Fig. 4B). Plastic deposition rates are also associated with human population increase in Santa Barbara, Ventura, Los Angeles, Kern, and San Luis Obispo counties (Pearson’s correlation, *r* = 0.81, *P* < 0.05), the counties that have the most direct effect on the Santa Barbara Basin watershed.

## DISCUSSION

The close correlation between plastic deposition and worldwide plastic production suggests a direct link between the exponential increase in plastic production and consumption and its effect on ocean ecosystems. Our results demonstrate that such an increase is now detectable not only in surface ocean water but also in a benthic ecosystem, as recorded in the sedimentary record. This result calls for limiting the plastic waste stream that enters the ocean, because plastic sedimentation is directly mirroring the ever-increasing production trends.

The close relationship between the increase in coastal population of the Santa Barbara drainage basin and plastic deposition agrees with Browne *et al*. (*2*) and Eriksen *et al*. (*14*), who both found more marine microdebris in areas of higher population density. However, those studies used current spatial patterns in population to infer the effects of population increase on microplastic abundance, while our study appears to be the first to analyze continuous temporal trends. This tight coupling leads us to predict that this growing rate of plastic deposition will continue to increase in the future, barring marked changes in policy or waste management.

The core was sieved through a 104-μm mesh, establishing the minimum size of detectable particles. The vast majority of particles were between 500 and 1000 μm in length, and this trend was consistent throughout the core, which could be related to visually sorting the core and perhaps missing some smaller particles (fig. S2). But it is noteworthy that these prevalent particles, from 104 to 1000 μm, are some of the smallest recorded particles in any sediment study (*15*) and show that small microplastics are making their way to the marine benthos where they are likely to be ingested by benthic deposit feeders.

This deposited microplastic can enter the benthic food web. Benthic animals have been shown to ingest and become entangled in plastic (*16*–*18*), and microplastics are being found in sediments, especially in urban, populated areas (*2*). The plastics entering the benthic food web not only are hydrocarbon chains but also can contain harmful additives and colorants (*19*) and can adsorb persistent organic pollutants like polycyclic aromatic hydrocarbons, polychlorinated biphenyls, and dichlorodiphenyltrichloroethane from the surrounding environment (*20*, *21*). These microplastics are ingested by small animals near the base of the food web and, along with their harmful additives, have the potential to accumulate in the food web, affecting much larger animals (*22*, *23*). Ingested plastics have been shown to cause liver toxicity (*21*) and brain damage to animals (*23*), among other ailments, and for these ecological and toxicological reasons, it is essential to understand how much plastic is in coastal sediments, how long it has been accumulating there, and pathways of incorporation into the food web.

In considering the limitations of this study, only one box core could be analyzed; however, we assume that the box core is representative of the variability in deposition rate over the basin, as evidenced by the close alignment of stratigraphic events between the Cal-Echoes sediment cores and SPR0901-06KC, the most recently and accurately dated Santa Barbara Basin sediment core (*6*, *24*). The box core was first sampled and analyzed for fish otoliths, and future cores should be analyzed with particular attention to limiting contamination, especially by microfibers, in all stages of sampling and subsampling. The use of a micro-FTIR would be helpful in future studies to identify the smallest particles.

### The Anthropocene

In calling for a new geological epoch, the Anthropocene, Zalasiewicz *et al*. (*9*) identified the need for more geological proxies in the sediment record to mark the change from pre-1945 to post-1945, the dawn of the “Great Acceleration” of modern civilization. Although they pinpointed the release of plutonium in New Mexico in 1945 as perhaps the easiest to identify, they suggested that plastic, especially in nonbioturbated sediment, would likely be a very useful stratigraphic marker (*9*, *10*). Although plastics have been found in surficial sediment samples (*25*, *26*) and even in a few select layers of cores showing a temporal increase (*27*), this is the first known study of a continuous survey in nonbioturbated sediment. After taking into account airborne and processing plastic contamination, our results identify this Great Acceleration by revealing a tightly coupled relationship between worldwide plastic production, regional population growth, and the plastic deposited in the sedimentary record, whose consequences for deposit-feeding benthic organisms and ocean food webs are poorly understood.

## MATERIALS AND METHODS

### Obtaining the sediment core

A box core was used to sample sediments from the Santa Barbara Basin off the coast of California in October 2010 during the Scripps Institution of Oceanography Cal-Echoes research cruise (fig. S1), at 34°17.228′N, 120°02.135′W, at a water depth of approximately 580 m. Site 1, the site of the box core MV1012-ST46.9-BC1 (BC1), was chosen as a reoccupation of Ocean Drilling Program Site 893.

### Imaging and dating the sediment core

Color photographs of the core were taken on the deck of the ship before subcoring. The box core was removed from the coring equipment on deck by subcoring with rectangular plastic core liners of 76 cm length and 15 cm width. The box core was subcored with only one plastic core liner. The subcore section was housed in the core liner and placed into Hybar trilaminate membrane bags with oxygen absorbers, flushed with nitrogen, vacuum-sealed, and stored at 4°C.

One thin vertical slab (2 cm thick) was trimmed off the side of each subcore section with a saw. Vertical core slabs were x-radiographed at the Scripps Institution of Oceanography Geological Collections using a Geotek MSCL-XR core scanner, which combined individual two-dimensional images to make the composite x-radiograph images. The core slabs were scanned at 1-mm intervals in a linear, nonrotational scan.

X-radiographs and color photographs were used to develop a high-resolution chronology for the core. Several age models have been developed to assign dates to the Santa Barbara Basin varved stratigraphy. The traditional age model relied on counting seasonal varve couplets and was used to establish a chronology for the top 35 cm of the box core (Fig. 1) (*28*).

### Sieving and drying the sediment core

The 76-cm subcore was cut transversely every 0.5 cm with very fine wire, and the location of each cut was recorded. Then, sediment samples were stored frozen before further processing. Transverse samples were oven-dried overnight at 50°C, washed, and then wet-sieved in metal sieves using a 104-μm mesh over a 65-μm mesh. The >104-μm fraction was first sorted under a dissecting microscope by W.J. for fish otoliths before being sorted for microplastics. Core chronology was resolved to the upper and lower edges of the 0.5-cm transverse samples; the dates assigned to the upper and lower edges were averaged and used to assign dates to samples found within the transverse section.

### Sampling the sediment core for plastics

The samples used in the present analysis were the >104-μm fraction from the box core. Samples were visually sorted under a Wild M5 dissecting microscope at 12× magnification for likely microplastic pieces, which were photographed with either a Canon Powershot S5 IS or Canon E0S Rebel T5i camera. Measurements were made to 66-μm resolution with a calibrated ocular micrometer.

Sediment was sorted in small aliquots on a black sediment sorting tray, with gridded squares. Sorted pieces of plastic were removed from the sediments, counted, measured for length with an ocular micrometer (fig. S2), and photographed for length and shape. In some cases, plastic fibers were so curved or twisted that a feret maximum length was measured instead of a true length. A description of the particles’ physical appearance, color, amount and location of fouling, and whether they were agglomerated with other particles was recorded. They were sorted into categories: Fiber, Film, Fragment, and Spherical (Fig. 2 and fig. S3). Sorted plastic pieces were stored in four-cavity paleontological slides with glass covers until later analysis by FTIR. Whenever sorting was not actively in progress, the sorting tray was covered to limit airborne contamination of microplastics from the laboratory space.

Plastic particles were initially visually differentiated from biological or sedimentary particles by their color and shape; the sediment was predominantly composed of foraminifera tests, shells, and striated biological film that appeared as if it were once living. A few shapes seemed to dominate the natural material in the sample. Plastic, in contrast, was predominately composed of elongate fibers or sharp-edged fragments that did not all look similar. It has been shown that brightly colored plastics are often overcounted in comparison to duller, more biological colored pieces of plastic (*2*), so the sediments were sorted against a black background to reduce that bias. Potential microspheres were examined under 50× magnification to assess whether the matrix of pores common to foraminifera tests could be discerned; often the matrix was visible on higher magnification, and the sphere was then deemed biological. If films had striations that made them look like they may have once been living (e.g., part of a molt), they were also not counted. In general, if a particle’s origin was in question, it was deemed part of the sediment and not removed for further FTIR analysis or counted as part of the plastic abundance numbers. To reduce sampling bias between multiple sorters, all images of plastic pieces were personally examined by the senior author to assess whether it was likely plastic or likely biological. Questionable images were removed from the plastic enumerations.

### Identification of plastics via FTIR spectroscopy

To determine whether visually identified plastics were plastic polymers, we analyzed a subset of particles using an FTIR spectrometer with an attenuated total reflectance diamond crystal attachment (Nicolet 6700 with Smart-iTR). All spectra were recorded at 4 cm^−1^ resolution. The FTIR spectra for particles collected from the ocean were compared to published standards (*2*, *15*, *29*, *30*) to attempt to identify whether the particles were plastic, biological material, or sedimentary material. The spectra were analyzed via eFTIR software (www.essentialFTIR.com). The plastic was further identified to plastic type when possible. At least 10% of particles from every fifth 0.5-cm transverse sediment layer from the box core were identified by FTIR. The trilaminate bag and core liner in which the core was stored were also tested via FTIR to identify sources of contamination.

### Plastic deposition rates

The box core had a surface area of approximately 174 cm^2^. To calculate plastic deposition rates, the number of plastic particle pieces in a 0.5-cm transverse layer was divided by the time interval represented by the transverse layer and normalized to 100 cm^2^ of seafloor during 1 year (No. particles*100 cm^−2^ year^−1^). The plastic deposition rate (Particles*100 cm^−2^ year^−1^) was calculated for the four individual particle types, from 1836 to 2009 (fig. S4). Pieces of core liner, identified via FTIR, were treated separately from other fragments. Fibers dominated the fluxes (fig. S4B).

The deposition rate of particles in layers corresponding to 1836–1945 was averaged, as an indication of baseline contamination of plastic particles. The contamination values before 1945 were relatively consistent throughout the 1836–1945 region (left of the gray line in fig. S4), and thus, an averaging is appropriate here. Any plastic before 1945 was treated as contamination due to the low amounts of plastic in production at that time (*7*), and the fact that some plastics (PET, polyester, polypropylene, etc.) had not yet been invented. The bottom sample of the core, corresponding to 1834, was removed from analysis due to previous knowledge that it had high levels of contamination from contact with the bottom of the box core liner during processing. The average from 1836 to 1945 was then subtracted from the deposition rate of all other samples to correct for baseline contamination.

Baseline-corrected deposition rates from 1945 to 2009 were plotted against time, and an exponential was fitted. Residuals of plastic deposition rates were calculated from the exponential function (Fig. 4A). Deposition rates were calculated for fragments, film, and spherical particles (fig. S6A) separate of fibers (fig. S6B), and both had an exponential increase, with no significant difference between them (ANCOVA, *P* > 0.05).

### Plastic deposition rates and environmental factors

The residuals of plastic deposition from the exponential fit were compared to the residuals of rainfall for the urban watershed that feeds into the Santa Barbara Basin (fig. S5). Rainfall records from downtown Los Angeles and downtown Santa Barbara were recorded annually for 1 July to 30 June 1877–2017 for Los Angeles (*11*) and September to August 1899–2017 for Santa Barbara (*12*). Residuals from the 50-year means of rainfall data were compared to the residuals of plastic deposition. The residuals of plastic deposition were also compared to the ONI (*13*) for correlation with the El Niño–Southern Oscillation.

### Plastic deposition rates and societal trends

The southern California coastal population (Santa Barbara, Ventura, Kern, Los Angeles, and San Luis Obispo counties) from 1950 to 2010 (*31*, *32*) and worldwide plastic production from 1950 to 2010 (*8*) were also compared to plastic deposition rates (Fig. 4B).

### Statistical analysis

The relationship between plastic deposition and world plastic production in Fig. 4B was calculated using Pearson’s product-moment correlation and found to be significant (*P* < 0.01). The relationships between rainfall residuals and 50-year means of rainfall and the ONI (fig. S5) were calculated using Pearson’s correlation and found to be not significant (*P* > 0.05). The exponential growth curves in Fig. 4A and fig. S6 were fitted to a simple exponent with two parameters and showed a significant fit (*P* < 0.001). The post-1945 rate of increase of combined fragments, films, and spherical particles (fig. S6A) was compared to the rate of accumulation of fibers alone (fig. S6B), using an ANCOVA test, and was found to be not significantly different (*P* > 0.05).

## Supplementary Material

http://advances.sciencemag.org/cgi/content/full/5/9/eaax0587/DC1

Download PDF

Multidecadal increase in plastic particles in coastal ocean sediments

## SUPPLEMENTARY MATERIALS

Supplementary material for this article is available at http://advances.sciencemag.org/cgi/content/full/5/9/eaax0587/DC1 Supplementary Text Fig. S1. Santa Barbara Basin bathymetry and sampling locations. Fig. S2. Size distribution of sampled particles. Fig. S3. Distribution of sampled particle types. Fig. S4. Deposition rates of individual plastic types over time, 1836–2009. Fig. S5. Plastic deposition and weather residuals. Fig. S6. Deposition rates of plastic types once contamination value removed. Table S1. FTIR spectroscopy survey of box core. References (*33*–*40*)
