# Supplementary material for: Multidecadal increase in plastic particles in coastal ocean sediments
Source: Sci Adv. 2019 Sep 4;5(9):eaax0587. doi: 10.1126/sciadv.aax0587 (PMC6726453; doi:10.1126/sciadv.aax0587)
Supplement: http://advances.sciencemag.org/cgi/content/full/5/9/eaax0587/DC1 [file supp_5_9_eaax0587__index.html]

Science Advances | Science AdvancesAAASSearchScience AdvancesMenu

## Supplementary Materials

**This PDF file includes:**

- Supplementary Text
- Fig. S1. Santa Barbara Basin bathymetry and sampling locations.
- Fig. S2. Size distribution of sampled particles.
- Fig. S3. Distribution of sampled particle types.
- Fig. S4. Deposition rates of individual plastic types over time, 1836-2009.
- Fig. S5. Plastic deposition and weather residuals.
- Fig. S6. Deposition rates of plastic types once contamination value removed.
- Table S1. FTIR spectroscopy survey of box core.
- References (*33*–*40*)

Download PDF

**Files in this Data Supplement:**

- Adobe PDF - aax0587\_SM.pdf
