# Supplementary material for: Multidecadal increase in plastic particles in coastal ocean sediments
Source: Sci Adv. 2019 Sep 4;5(9):eaax0587. doi: 10.1126/sciadv.aax0587 (PMC6726453; doi:10.1126/sciadv.aax0587)
Supplement: Download PDF [file aax0587_SM.pdf]

Science Advances
AAAS

advances.sciencemag.org/cgi/content/full/5/9/eaax0587/DC1

Supplementary Materials for

Multidecadal increase in plastic particles in coastal ocean sediments

Jennifer A. Brandon*, William Jones, Mark D. Ohman

*Corresponding author. Email: jabrandon@ucsd.edu

Published 4 September 2019, *Sci. Adv.* **5**, eaax0587 (2019)
DOI: 10.1126/sciadv.aax0587

This PDF file includes:

Supplementary Text
Fig. S1. Santa Barbara Basin bathymetry and sampling locations.
Fig. S2. Size distribution of sampled particles.
Fig. S3. Distribution of sampled particle types.
Fig. S4. Deposition rates of individual plastic types over time, 1836–2009.
Fig. S5. Plastic deposition and weather residuals.
Fig. S6. Deposition rates of plastic types once contamination value removed.
Table S1. FTIR spectroscopy survey of box core.
References (*33–40*)

Supplementary Text

1.1 Obtaining the sediment core

We assume that this single sediment core is representative of the variability in deposition rate over the basin. The close alignment of stratigraphic events between the Cal-ECHOES sediment cores and SPR0901-06KC, the most recently and accurately dated SBB sediment core, indicates similar conditions among cores (*6*, *24*).

1.2 Imaging and dating the sediment core

Hendy et al. (2013)(*6*) and Schimmelmann et al. (2013)(*24*) used ^{14}C dates from planktonic foraminiferal carbonate and terrestrial-derived organic carbon from Kasten core SPR0901-06KC to show that accuracy of the traditional varve counting method decreases prior to approximately 1700 AD due to under-counting of varves. The present box core was sufficiently shallow that it did not necessitate this correction.

1.3 Identification of plastics via FTIR spectroscopy

Overall, FTIR permitted definitive identification of 53.0% of the subsampled visually identified microplastics to specific plastic polymer type, and confirmation of an additional 34.5% as most likely plastic (Fig. 3, table S1). Column 5, table S1 equals the 53.0% of definitively identified plastics. The additional 34.5% equals Column 4 minus Column 5 values, since every particle identified as most likely plastic is in Column 4, and a subset of those values that were definitively identified is in Column 5.

Subsampling was done by the senior author at the time of FTIR analysis. This was done visually, by removing identified particles by forceps from their sample slides, and placing them on the FTIR. At least 10% of each transect layer was analyzed this way, with every

effort to mimic the proportions of plastics collected (i.e., sampling more fibers than fragments, film, or spherical particles due to their higher presence in core, fig. S4). There is most likely some selection bias in the subsampling, where particles were so small that the senior author could not see them or properly transfer them for FTIR analysis. There is also slight overrepresentation of fragments and film particles (table S1, Column 3), because they are easier to analyze via FTIR than fibers.

Some of the particles that could not be definitively identified to plastic type had spectra that looked similar to two plastic types that could not be differentiated. This problem was common with particles that showed the triplicate diagnostic peak of polyvinyl chloride (PVC) and polyethylene terephthalate (PET) and particles that showed the doublet peak of polyethylene (PE) and polystyrene (PS), but could not be differentiated further, either because of the quality of the spectra, particle weathering that occluded differentiating regions of the spectra (*33*), or because the real-world plastic particles contained additives, colorants, etc. that are known to change the shape of FTIR spectra from pure standards (*34*). LDPE was differentiated from HDPE by the presence of a small peak at 1377 cm^{-1}, and if its presence/absence was not clear from the spectra, the piece was recorded as PE (*33*).

The particles that could not be identified (table S1, Column 6) were often too small or thin to generate good FTIR spectra or they had readings that were not conclusive. Some of those particles were visually recorded at the time of FTIR reading as “thin white fiber” or “reddish black fragment,” so they are likely plastic, but this cannot be confirmed. A few particles had clean spectra similar to the spectra of calcium carbonate, aragonite, or clay (table S1, Column 7)(*29*). These particles were visually recorded at the time of FTIR

reading as “filmy fragment,” “tiny off-white fragment,” “fragment/sediment, looks biological,” or “really thin fiber with sediment still stuck to it.” It is likely that these particles were organic or sedimentary, or in the last case, had sediment still attached to them. Particles that appeared organic or sedimentary under microscopy were already removed from the plastic enumerations during the secondary proofing of the images by the senior author. However, these particles were removed from the plastic abundance calculations but were not physically removed from the paleontological sorting tray. Thus, these particles that were subsampled and analyzed by FTIR (table S1), and had organic or sedimentary signatures, had already been excluded from the plastic abundance enumerations.

1.4 Plastics as contamination

Any plastic before 1945 was treated as contamination due to the low amounts of plastic in production at that time (*7*). However, this procedure could overestimate contamination, as synthetic plastic was invented in 1907 (*7*) and did exist in small amounts before World War II (*7*). But we assume nearly all of these pieces were added during core processing.

The trilaminate bag in which the core was stored was PET, as identified by FTIR, and any contamination from the bag could not be differentiated from other PET unless the fragments were large enough to determine whether they were metallic silver (like the trilaminate bag) or another color (Fig. 3B). However the bag was removed before the core was cut, and so any trilaminate bag contamination would be incidental. Modest contamination was found from the plastic core liner box that was cut with a saw to make the core chronology; small numbers of un-aged, identical fragments of that unique spectrum were found intermittently throughout the core (Fig. 3E; fig. S4B). Those

fragments were included in Column 4 and 5 of table S1, because these fragments were known to be plastic and identified to type (type = core liner, because its unique FTIR spectrum is not identical to any published standard). These fragments were obviously contamination, and were thus accounted for in the baseline contamination subtraction.

We attempted to use the spectral signatures of plastic degradation (*33*) to assess which pieces were aged and thus more likely to be contamination. However, a marked temporal trend in aging was not seen between layers of the core denoting contamination and non-contamination plastics.

1.5 Fiber contamination and deposition

The majority of plastics found in the core overall were fibers, including in the contamination samples, from 1834-1945, where 89.1% of the samples were fibers (fig. S3). This result agrees with both Browne et al. (2011)(*2*) and Thompson et al. (2004)(*35*) who found high densities of fibers in sediments, but also Foekema et al. (2013)(*36*) and Davison and Asch (2011)(*37*) who found fibers as sources of contamination in their samples. Because of previous research such as Foekema et al. (2013) and Davison and Asch (2011), we hypothesized that most of the contamination would be airborne fibers, as was found. But the fact that the core spans years before and after the prevalence of plastic allows us to subtract the contamination values and determine that there are still numerous fibers buried in the sediment itself, which agrees with earlier literature (*2*, *35*).

1.6 Plastic fragments and spheres

Fourteen percent of the particles in the core were plastic fragments, although many more in the post-1945 samples than pre-1945 samples (fig. S3). Almost no spherical plastics

were found in the core, despite the fact that microbeads in consumer products have a diameter of ~400-500 µm and would have been retained in the 104 µm sieve (*14*, *38*). One of the only perfectly spherical microbeads we found was an un-aged polystyrene microbead in an early sample (circa 1893, before the advent of plastic) and was thus contamination.

1.7 Buoyancy of plastics

PE is less dense than seawater and would not logically be found in the sediment; however we found multiple pieces of it. One piece of plastic was also identified as most likely polypropylene (PP), which is also less dense than seawater. However, Browne et al. (2011)(*2*) also found polypropylene and polyethylene in sediment samples near areas of sewage and wastewater effluent, and it is likely that our samples contain particles from the effluent and riverine flow off the Santa Barbara coastline. These buoyant plastics could have also sunk to the bottom through fecal or marine snow transport (*22*, *39*), or via biofouling (*40*). It is also possible that these buoyant pieces were airborne or processing contamination.

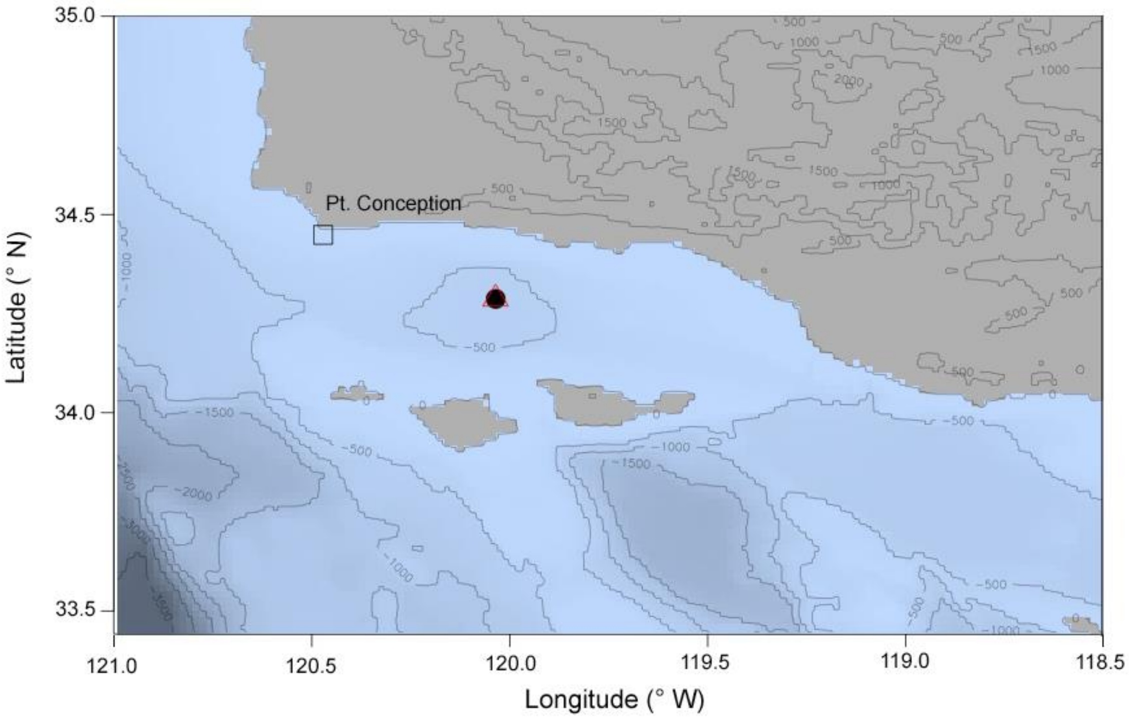

Fig. S1. Santa Barbara Basin bathymetry and sampling locations. Box core 1 was collected from Site 1 (black circle; 34.387133, 120.035583; 580 m water depth). Location of Ocean Drilling Program Site 893 used in chronology development also shown (red triangle; 34.2875, -120.036; 577 m water depth).

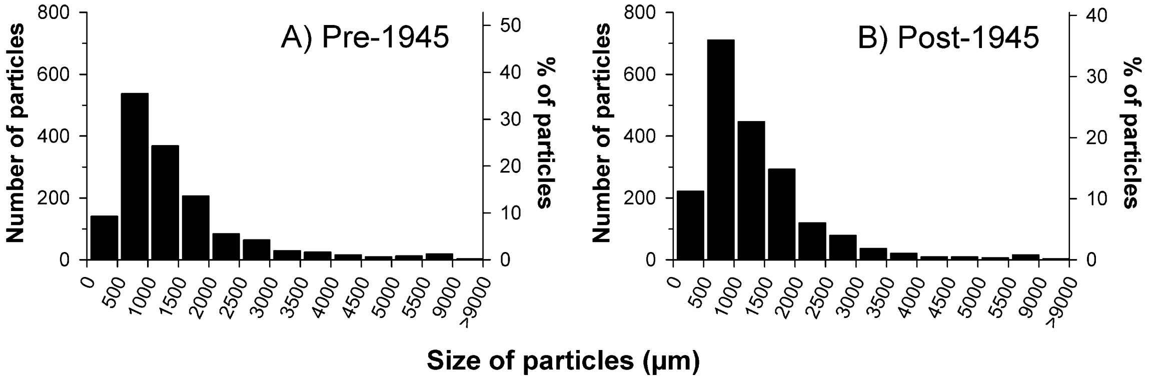

Fig. S2. Size distribution of sampled particles. Size distribution of particles in layers A) before and B) after 1945. X axis: upper bound of size bin (μm).

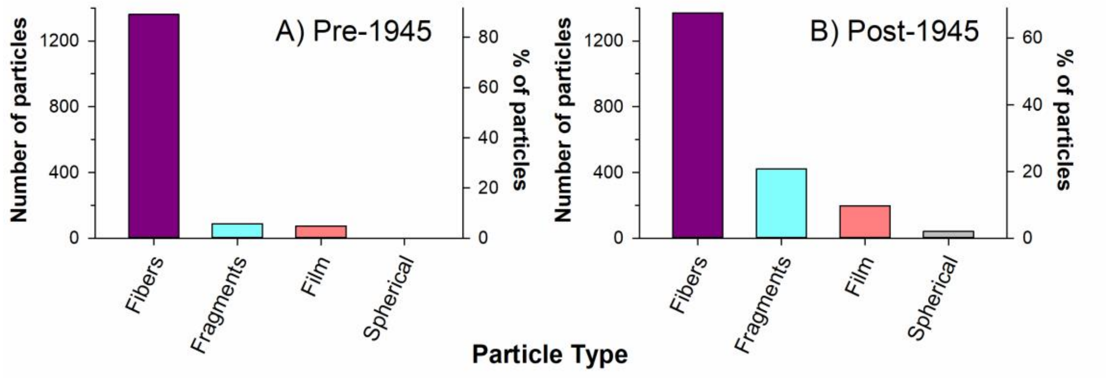

Fig. S3. Distribution of sampled particle types. Particle types in the core A) before and B) after 1945 (by total number of particles).

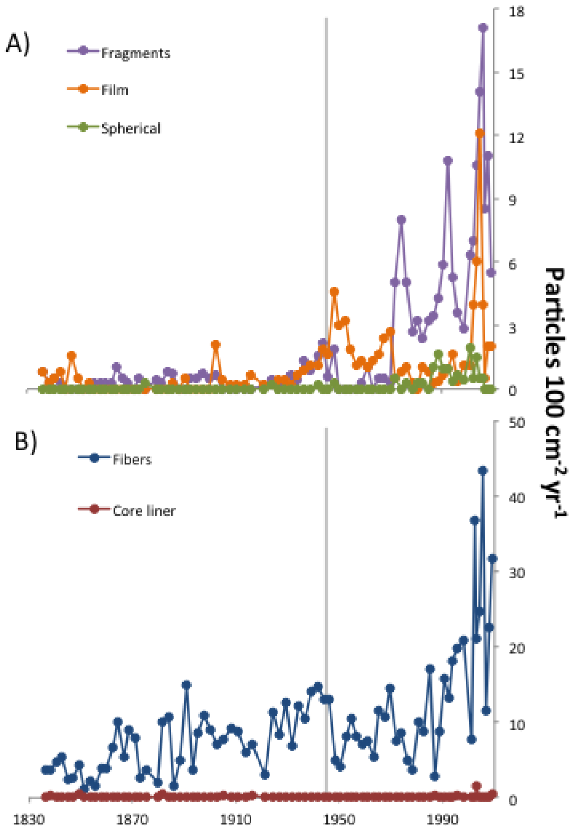

Fig. S4. Deposition rates of individual plastic types over time, 1836–2009. Plastic deposition rates in seabed sediment, 1836-2009, by individual particle types: A) Fragments, film, and spherical particles; B) Fibers and core liner. Gray line indicates 1945. All data to the left of line considered contamination, all data to the right of line not adjusted for possible contamination here.

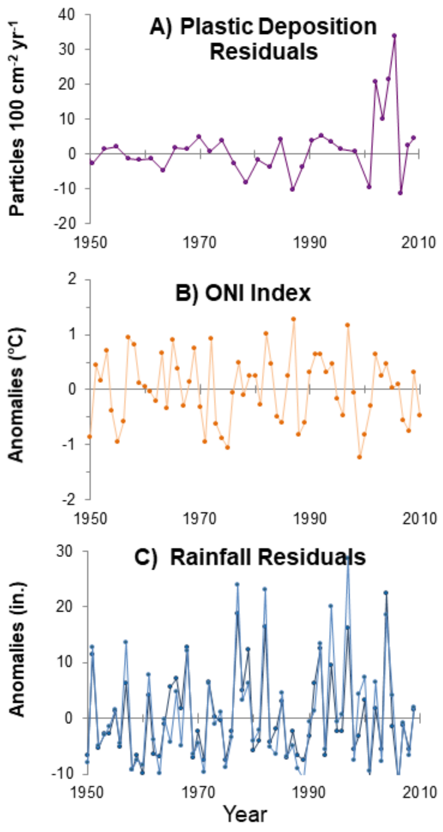

Fig. S5. Plastic deposition and weather residuals. A) Plastic residuals calculated from exponential fit of all particles. B) ONI (Oceanic Niño Index) residuals calculated from yearly averages. C) Rainfall residuals calculated from 50-year mean. Dark blue: Santa Barbara County rainfall. Light blue: Los Angeles County rainfall. Data from A not correlated with data from B or C ($p > 0.05$).

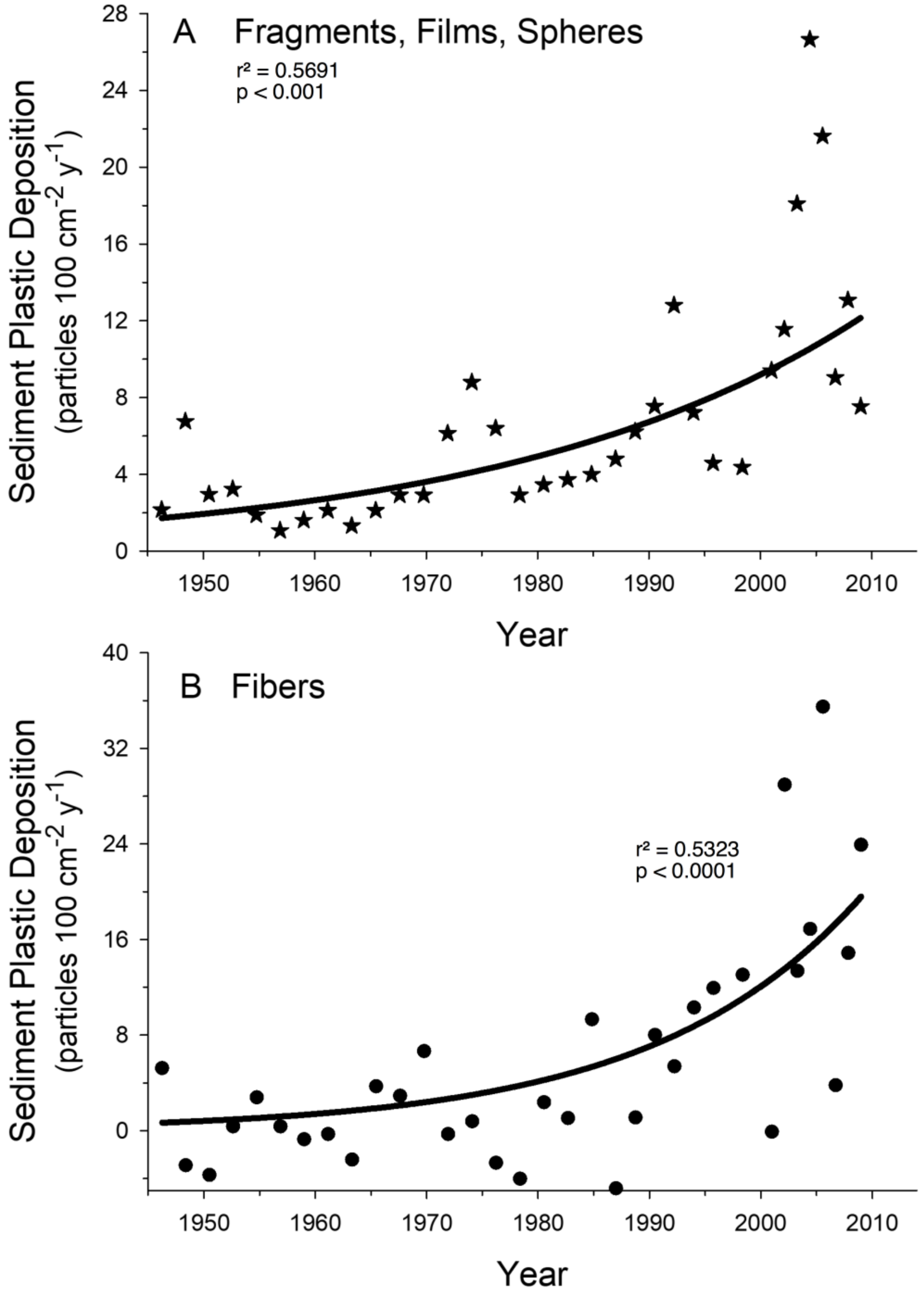

Fig. S6. Deposition rates of plastic types once contamination value removed. Particle deposition rates, 1945-2009, minus contamination value; A) Fragments, film, and spherical particles deposition rates, B) fiber deposition rate.

Table S1. FTIR spectroscopy survey of box core.

Layer number (Year)	Number (%) of particles sampled for FTIR	Ratio of Fibers: Film: Fragments : Spherical	Particles likely plastic	Plastics identified to type	Bad reads	May be sediment
**1 (2009.0)**	9 (11.5%)	5:3:1:0	5	2	4	0
**6 (2003.3)**	10 (13.0%)	4:2:4:0	9	3	1	0
**11 (1995.8)**	8 (10.8%)	3:1:4:0	8	3	0	0
**16 (1987.0)**	7 (24.1%)	1:1:5:0	5	2	2	0
**21 (1976.2)**	12 (48.8%)	9:0:3:0	12	5	0	0
**26 (1965.5)**	12 (23.5%)	8:3:1:0	11	8	1	0
**31 (1954.8)**	11 (23.9%)	8:2:1:0	7	3	4	0
**36 (1944.1)**	12 (19.0%)	6:2:4:0	10	8	1	1
**41 (1931.8)**	7 (20%)	6:0:1:0	6	3	0	1
**47 (1916.3)**	12 (33.3%)	10:0:2:0	12	7	0	0
**51 (1905.4)**	12 (31.6%)	8:1:3:0	12	5	0	0
**56 (1893.1)**	5 (29.4%)	3:0:1:1	4	4	1	0
**61 (1881.8)**	8 (20.5%)	5:1:2:0	8	5	0	0
**66 (1871.0)**	6 (20%)	5:0:1:0	6	5	0	0
**71 (1860.2)**	6 (37.5%)	3:0:3:0	6	5	0	0
**76 (1849.3)**	8 (44.4%)	5:1:2:0	8	5	0	0
**81 (1838.5)**	6 (37.5%)	5:0:1:0	4	4	0	2

Particles from every fifth transect layer measured via FTIR for spectral identification.
